# (*S*)-Perillaldehyde azine

**DOI:** 10.1107/S1600536810004071

**Published:** 2010-02-06

**Authors:** Li-Lu Han, Jiang-Hua Shi, De-Peng Yan, Seik Weng Ng

**Affiliations:** a Hunan Yongzhou Vocational College, Yongzhou, Hunan 425100, People’s Republic of China; b Department of Biology and Chemistry, Hunan University of Science and Engineering, Yongzhou, Hunan 425100, People’s Republic of China; c Department of Chemistry, University of Malaya, 50603 Kuala Lumpur, Malaysia

## Abstract

The C=N–N=C linkage [torsion angle −172.5 (2)°] in the title azine, C_20_H_28_N_2_, adopts a *trans* conformation. The six-membered rings adopt sofa conformations.

## Related literature

A previous study reported the oxime derivative of *S*-perillaldehyde; see Yuan *et al.* (2009 ▶). Only few crystal structures of azines have been reported, see: Berthou *et al.* (1970 ▶); Kim & Lee (2008 ▶); Marek *et al.* (1997 ▶); Rizal *et al.* (2008 ▶); Sanz *et al.* (1999 ▶).
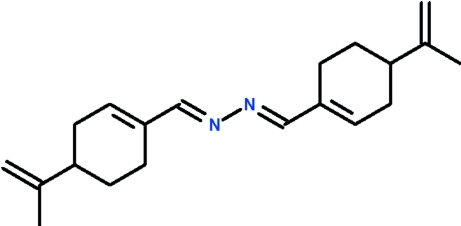


## Experimental

### 

#### Crystal data

C_20_H_28_N_2_
*M* *_r_* = 296.44
Monoclinic, 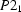

*a* = 8.8200 (5) Å
*b* = 9.7603 (6) Å
*c* = 10.1710 (6) Å
β = 94.970 (1)°
*V* = 872.29 (9) Å^3^
*Z* = 2
Mo *K*α radiation
μ = 0.07 mm^−1^
*T* = 173 K
0.48 × 0.46 × 0.21 mm

#### Data collection

Bruker SMART APEX diffractometer
7179 measured reflections
2013 independent reflections
1802 reflections with *I* > 2σ(*I*)
*R*_int_ = 0.035

#### Refinement

*R*[*F* ^2^ > 2σ(*F* ^2^)] = 0.046
*wR*(*F* ^2^) = 0.141
*S* = 1.12
2013 reflections
201 parameters
1 restraint
H-atom parameters constrained
Δρ_max_ = 0.22 e Å^−3^
Δρ_min_ = −0.23 e Å^−3^

### 

Data collection: *SMART* (Bruker, 2003 ▶); cell refinement: *SAINT* (Bruker, 2003 ▶); data reduction: *SAINT*; program(s) used to solve structure: *SHELXS97* (Sheldrick, 2008 ▶); program(s) used to refine structure: *SHELXL97* (Sheldrick, 2008 ▶); molecular graphics: *X-SEED* (Barbour, 2001 ▶); software used to prepare material for publication: *publCIF* (Westrip, 2010 ▶).

## Supplementary Material

Crystal structure: contains datablocks global, I. DOI: 10.1107/S1600536810004071/bt5184sup1.cif

Structure factors: contains datablocks I. DOI: 10.1107/S1600536810004071/bt5184Isup2.hkl

Additional supplementary materials:  crystallographic information; 3D view; checkCIF report

## Figures and Tables

**Fig. 1.**
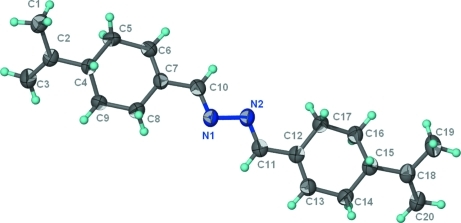
Anisotropic displacement ellipsoid plot (Barbour, 2001) of C20H28N2 at the 70% probability level; hydrogen atoms are shown as spheres of arbitrary radius.

## supplementary crystallographic information

### Experimental

An ethanol solution (10 ml) of hydrazinium hydroxide (0.5 g, 0.01 mol) was added to a 50% ethanol solution (50 ml) of perillaldehyde (3 g, 0.02 mol); acetic acid (2 ml) was then added. The mixture was heated for two hours. The product was recrystallized from ethyl acetate to afford light-yellow crystals (yield 70%).

### Refinement

Carbon-bound H-atoms were placed in calculated positions (C—H 0.95–1.00 Å) and were included in the refinement in the riding model approximation, with *U*(H) set to 1.2*U*(C). In the absence of anomalous scatterers Friedel pairs were merged. The chiral carbon atoms were assumed to have an *S*-configuration, i.e., the configuration of perillaldehyde itself.

### Crystal data

**Table:**

C_20_H_28_N_2_	*F*(000) = 324
*M**_r_* = 296.44	*D*_x_ = 1.129 Mg m^−^^3^
Monoclinic, *P*2_1_	Mo *K*α radiation, λ = 0.71073 Å
Hall symbol: P 2yb	Cell parameters from 4048 reflections
*a* = 8.8200 (5) Å	θ = 2.3–27.2°
*b* = 9.7603 (6) Å	µ = 0.07 mm^−^^1^
*c* = 10.1710 (6) Å	*T* = 173 K
β = 94.970 (1)°	Block, yellow
*V* = 872.29 (9) Å^3^	0.48 × 0.46 × 0.21 mm
*Z* = 2	

### Data collection

**Table:**

Bruker SMART APEX diffractometer	1802 reflections with *I* > 2σ(*I*)
Radiation source: fine-focus sealed tube	*R*_int_ = 0.035
graphite	θ_max_ = 27.2°, θ_min_ = 2.0°
ω scans	*h* = −11→11
7179 measured reflections	*k* = −12→12
2013 independent reflections	*l* = −12→12

### Refinement

**Table:**

Refinement on *F*^2^	Primary atom site location: structure-invariant direct methods
Least-squares matrix: full	Secondary atom site location: difference Fourier map
*R*[*F*^2^ > 2σ(*F*^2^)] = 0.046	Hydrogen site location: inferred from neighbouring sites
*wR*(*F*^2^) = 0.141	H-atom parameters constrained
*S* = 1.12	*w* = 1/[σ^2^(*F*_o_^2^) + (0.074*P*)^2^ + 0.242*P*] where *P* = (*F*_o_^2^ + 2*F*_c_^2^)/3
2013 reflections	(Δ/σ)_max_ = 0.001
201 parameters	Δρ_max_ = 0.22 e Å^−^^3^
1 restraint	Δρ_min_ = −0.23 e Å^−^^3^

### Fractional atomic coordinates and isotropic or equivalent isotropic displacement parameters (Å^2^)

**Table:**

	*x*	*y*	*z*	*U*_iso_*/*U*_eq_	
N1	1.1573 (3)	0.4999 (3)	0.9681 (2)	0.0344 (5)	
N2	1.2864 (3)	0.5570 (3)	1.0416 (2)	0.0340 (5)	
C1	0.3418 (3)	0.5570 (4)	0.6676 (3)	0.0432 (7)	
H1A	0.2556	0.5311	0.6053	0.065*	
H1B	0.3581	0.6561	0.6631	0.065*	
H1C	0.3200	0.5317	0.7573	0.065*	
C2	0.4821 (3)	0.4836 (3)	0.6326 (3)	0.0335 (6)	
C3	0.4827 (4)	0.4131 (4)	0.5213 (3)	0.0451 (8)	
H3A	0.3933	0.4085	0.4623	0.054*	
H3B	0.5725	0.3672	0.5006	0.054*	
C4	0.6201 (3)	0.4954 (3)	0.7323 (3)	0.0303 (6)	
H4	0.5881	0.4606	0.8182	0.036*	
C5	0.6657 (4)	0.6461 (3)	0.7542 (3)	0.0403 (7)	
H5A	0.6717	0.6907	0.6674	0.048*	
H5B	0.5856	0.6933	0.7995	0.048*	
C6	0.8147 (3)	0.6631 (3)	0.8341 (3)	0.0369 (6)	
H6	0.8407	0.7518	0.8673	0.044*	
C7	0.9134 (3)	0.5613 (3)	0.8618 (3)	0.0302 (6)	
C8	0.8822 (3)	0.4179 (3)	0.8139 (3)	0.0373 (7)	
H8A	0.8524	0.3612	0.8882	0.045*	
H8B	0.9767	0.3785	0.7837	0.045*	
C9	0.7568 (3)	0.4114 (3)	0.7014 (3)	0.0356 (6)	
H9A	0.7962	0.4465	0.6196	0.043*	
H9B	0.7255	0.3149	0.6860	0.043*	
C10	1.0558 (3)	0.5917 (3)	0.9402 (3)	0.0325 (6)	
H10	1.0739	0.6824	0.9716	0.039*	
C11	1.3954 (3)	0.4699 (3)	1.0567 (3)	0.0321 (6)	
H11	1.3816	0.3810	1.0195	0.038*	
C12	1.5398 (3)	0.5041 (3)	1.1296 (3)	0.0302 (6)	
C13	1.6523 (3)	0.4117 (3)	1.1373 (3)	0.0340 (6)	
H13	1.6335	0.3259	1.0948	0.041*	
C14	1.8062 (3)	0.4349 (3)	1.2089 (3)	0.0349 (6)	
H14A	1.8814	0.4500	1.1436	0.042*	
H14B	1.8370	0.3517	1.2603	0.042*	
C15	1.8082 (3)	0.5578 (3)	1.3023 (3)	0.0318 (6)	
H15	1.7472	0.5319	1.3769	0.038*	
C16	1.7255 (3)	0.6772 (3)	1.2292 (3)	0.0370 (7)	
H16A	1.7332	0.7600	1.2855	0.044*	
H16B	1.7748	0.6974	1.1475	0.044*	
C17	1.5580 (3)	0.6425 (3)	1.1939 (3)	0.0384 (7)	
H17A	1.5035	0.6437	1.2750	0.046*	
H17B	1.5116	0.7131	1.1331	0.046*	
C18	1.9656 (3)	0.5985 (3)	1.3620 (3)	0.0342 (6)	
C19	1.9691 (4)	0.7035 (4)	1.4708 (3)	0.0460 (8)	
H19A	2.0750	0.7240	1.5018	0.069*	
H19B	1.9160	0.6674	1.5442	0.069*	
H19C	1.9186	0.7875	1.4373	0.069*	
C20	2.0927 (3)	0.5473 (4)	1.3233 (3)	0.0407 (7)	
H20A	2.1881	0.5766	1.3641	0.049*	
H20B	2.0888	0.4812	1.2546	0.049*	

### Atomic displacement parameters (Å^2^)

**Table:**

	*U*^11^	*U*^22^	*U*^33^	*U*^12^	*U*^13^	*U*^23^
N1	0.0284 (11)	0.0386 (13)	0.0355 (12)	−0.0022 (11)	−0.0012 (9)	−0.0030 (11)
N2	0.0286 (12)	0.0386 (13)	0.0342 (11)	−0.0017 (11)	−0.0011 (9)	−0.0028 (11)
C1	0.0339 (15)	0.0515 (18)	0.0434 (15)	0.0057 (15)	−0.0007 (12)	−0.0029 (15)
C2	0.0291 (13)	0.0356 (15)	0.0352 (13)	−0.0007 (13)	−0.0007 (11)	0.0034 (12)
C3	0.0401 (16)	0.052 (2)	0.0420 (17)	0.0017 (16)	−0.0050 (13)	−0.0098 (16)
C4	0.0303 (13)	0.0304 (13)	0.0297 (12)	0.0008 (12)	−0.0003 (10)	−0.0007 (11)
C5	0.0366 (16)	0.0284 (14)	0.0547 (17)	0.0077 (13)	−0.0036 (13)	−0.0055 (14)
C6	0.0350 (15)	0.0281 (14)	0.0466 (15)	−0.0013 (12)	−0.0019 (12)	−0.0054 (13)
C7	0.0287 (13)	0.0328 (14)	0.0289 (12)	−0.0008 (12)	0.0010 (10)	−0.0031 (11)
C8	0.0349 (15)	0.0314 (15)	0.0438 (15)	0.0055 (13)	−0.0064 (12)	−0.0025 (14)
C9	0.0356 (14)	0.0275 (14)	0.0426 (15)	0.0031 (12)	−0.0031 (12)	−0.0060 (13)
C10	0.0316 (14)	0.0328 (15)	0.0334 (13)	−0.0029 (12)	0.0038 (11)	−0.0045 (11)
C11	0.0336 (14)	0.0330 (16)	0.0296 (13)	−0.0038 (12)	0.0029 (10)	0.0011 (11)
C12	0.0299 (13)	0.0320 (14)	0.0288 (12)	−0.0032 (12)	0.0037 (10)	0.0027 (11)
C13	0.0343 (14)	0.0320 (14)	0.0349 (13)	−0.0020 (13)	−0.0015 (11)	0.0011 (12)
C14	0.0309 (14)	0.0318 (15)	0.0411 (15)	0.0020 (12)	−0.0027 (11)	0.0006 (12)
C15	0.0290 (13)	0.0362 (15)	0.0302 (12)	−0.0040 (12)	0.0027 (10)	0.0031 (12)
C16	0.0342 (15)	0.0318 (15)	0.0434 (15)	−0.0035 (12)	−0.0054 (12)	−0.0012 (12)
C17	0.0335 (15)	0.0349 (16)	0.0451 (16)	0.0027 (13)	−0.0061 (12)	−0.0021 (14)
C18	0.0351 (15)	0.0372 (16)	0.0296 (13)	−0.0048 (12)	−0.0011 (11)	0.0054 (11)
C19	0.0404 (17)	0.059 (2)	0.0382 (16)	−0.0084 (16)	0.0010 (12)	−0.0103 (15)
C20	0.0319 (15)	0.0471 (18)	0.0416 (15)	−0.0016 (14)	−0.0048 (12)	0.0004 (15)

### Geometric parameters (Å, °)

**Table:**

N1—C10	1.280 (4)	C10—H10	0.9500
N1—N2	1.421 (3)	C11—C12	1.456 (4)
N2—C11	1.282 (4)	C11—H11	0.9500
C1—C2	1.499 (4)	C12—C13	1.338 (4)
C1—H1A	0.9800	C12—C17	1.503 (4)
C1—H1B	0.9800	C13—C14	1.501 (4)
C1—H1C	0.9800	C13—H13	0.9500
C2—C3	1.325 (4)	C14—C15	1.529 (4)
C2—C4	1.519 (4)	C14—H14A	0.9900
C3—H3A	0.9500	C14—H14B	0.9900
C3—H3B	0.9500	C15—C18	1.519 (4)
C4—C9	1.513 (4)	C15—C16	1.533 (4)
C4—C5	1.537 (4)	C15—H15	1.0000
C4—H4	1.0000	C16—C17	1.528 (4)
C5—C6	1.493 (4)	C16—H16A	0.9900
C5—H5A	0.9900	C16—H16B	0.9900
C5—H5B	0.9900	C17—H17A	0.9900
C6—C7	1.335 (4)	C17—H17B	0.9900
C6—H6	0.9500	C18—C20	1.318 (4)
C7—C10	1.459 (4)	C18—C19	1.507 (4)
C7—C8	1.500 (4)	C19—H19A	0.9800
C8—C9	1.523 (4)	C19—H19B	0.9800
C8—H8A	0.9900	C19—H19C	0.9800
C8—H8B	0.9900	C20—H20A	0.9500
C9—H9A	0.9900	C20—H20B	0.9500
C9—H9B	0.9900		
			
C10—N1—N2	110.8 (3)	C7—C10—H10	119.0
C11—N2—N1	111.2 (2)	N2—C11—C12	121.5 (3)
C2—C1—H1A	109.5	N2—C11—H11	119.3
C2—C1—H1B	109.5	C12—C11—H11	119.3
H1A—C1—H1B	109.5	C13—C12—C11	119.1 (3)
C2—C1—H1C	109.5	C13—C12—C17	122.0 (3)
H1A—C1—H1C	109.5	C11—C12—C17	118.9 (3)
H1B—C1—H1C	109.5	C12—C13—C14	124.2 (3)
C3—C2—C1	121.0 (3)	C12—C13—H13	117.9
C3—C2—C4	123.2 (3)	C14—C13—H13	117.9
C1—C2—C4	115.8 (3)	C13—C14—C15	112.4 (2)
C2—C3—H3A	120.0	C13—C14—H14A	109.1
C2—C3—H3B	120.0	C15—C14—H14A	109.1
H3A—C3—H3B	120.0	C13—C14—H14B	109.1
C9—C4—C2	115.3 (2)	C15—C14—H14B	109.1
C9—C4—C5	110.2 (2)	H14A—C14—H14B	107.9
C2—C4—C5	110.7 (2)	C18—C15—C14	114.5 (2)
C9—C4—H4	106.7	C18—C15—C16	112.0 (2)
C2—C4—H4	106.7	C14—C15—C16	108.4 (2)
C5—C4—H4	106.7	C18—C15—H15	107.2
C6—C5—C4	113.1 (2)	C14—C15—H15	107.2
C6—C5—H5A	108.9	C16—C15—H15	107.2
C4—C5—H5A	108.9	C17—C16—C15	110.8 (2)
C6—C5—H5B	108.9	C17—C16—H16A	109.5
C4—C5—H5B	108.9	C15—C16—H16A	109.5
H5A—C5—H5B	107.8	C17—C16—H16B	109.5
C7—C6—C5	124.0 (3)	C15—C16—H16B	109.5
C7—C6—H6	118.0	H16A—C16—H16B	108.1
C5—C6—H6	118.0	C12—C17—C16	111.4 (3)
C6—C7—C10	118.6 (3)	C12—C17—H17A	109.3
C6—C7—C8	121.9 (2)	C16—C17—H17A	109.3
C10—C7—C8	119.5 (3)	C12—C17—H17B	109.3
C7—C8—C9	112.5 (2)	C16—C17—H17B	109.3
C7—C8—H8A	109.1	H17A—C17—H17B	108.0
C9—C8—H8A	109.1	C20—C18—C19	120.9 (3)
C7—C8—H8B	109.1	C20—C18—C15	123.6 (3)
C9—C8—H8B	109.1	C19—C18—C15	115.6 (3)
H8A—C8—H8B	107.8	C18—C19—H19A	109.5
C4—C9—C8	111.2 (2)	C18—C19—H19B	109.5
C4—C9—H9A	109.4	H19A—C19—H19B	109.5
C8—C9—H9A	109.4	C18—C19—H19C	109.5
C4—C9—H9B	109.4	H19A—C19—H19C	109.5
C8—C9—H9B	109.4	H19B—C19—H19C	109.5
H9A—C9—H9B	108.0	C18—C20—H20A	120.0
N1—C10—C7	122.1 (3)	C18—C20—H20B	120.0
N1—C10—H10	119.0	H20A—C20—H20B	120.0
			
C10—N1—N2—C11	−172.5 (2)	N1—N2—C11—C12	−179.6 (2)
C3—C2—C4—C9	−4.6 (4)	N2—C11—C12—C13	−176.9 (3)
C1—C2—C4—C9	175.2 (3)	N2—C11—C12—C17	3.0 (4)
C3—C2—C4—C5	121.3 (4)	C11—C12—C13—C14	179.7 (2)
C1—C2—C4—C5	−58.9 (4)	C17—C12—C13—C14	−0.2 (4)
C9—C4—C5—C6	−42.0 (3)	C12—C13—C14—C15	15.4 (4)
C2—C4—C5—C6	−170.7 (2)	C13—C14—C15—C18	−171.5 (2)
C4—C5—C6—C7	12.8 (4)	C13—C14—C15—C16	−45.7 (3)
C5—C6—C7—C10	179.6 (3)	C18—C15—C16—C17	−168.8 (2)
C5—C6—C7—C8	−0.1 (5)	C14—C15—C16—C17	63.9 (3)
C6—C7—C8—C9	17.4 (4)	C13—C12—C17—C16	16.9 (4)
C10—C7—C8—C9	−162.3 (2)	C11—C12—C17—C16	−163.0 (2)
C2—C4—C9—C8	−173.8 (3)	C15—C16—C17—C12	−48.7 (3)
C5—C4—C9—C8	60.0 (3)	C14—C15—C18—C20	9.7 (4)
C7—C8—C9—C4	−47.3 (4)	C16—C15—C18—C20	−114.2 (3)
N2—N1—C10—C7	178.1 (2)	C14—C15—C18—C19	−170.4 (3)
C6—C7—C10—N1	−178.4 (3)	C16—C15—C18—C19	65.7 (3)
C8—C7—C10—N1	1.2 (4)		
